# Weight and sleep health in OSA: exploring their link

**DOI:** 10.3389/frsle.2026.1828583

**Published:** 2026-05-18

**Authors:** Caroline J. Beatty, Shane A. Landry, Dwayne L. Mann, Simon A. Joosten, Kaitlin Day, Maxine P. Bonham, Denise M. O'Driscoll, Alan Young, Ladan Ghazi, Chiara Murgia, Terry P. Haines, Garun S. Hamilton, Bradley A. Edwards

**Affiliations:** 1 Department of Physiology, Biomedicine Discovery Institute, Monash University, Melbourne, VIC, Australia; 2 School of Psychological Sciences and Turner Institute for Brain and Mental Health, Monash University, Melbourne, VIC, Australia; 3 School of Electrical Engineering and Computer Science, The University of Queensland, Brisbane, QLD, Australia; 4 Monash Lung, Sleep, Allergy and Immunology, Monash Health, Melbourne, VIC, Australia; 5 School of Clinical Sciences, Monash University, Melbourne, VIC, Australia; 6 Department of Nutrition, Dietetics and Food, School of Clinical Sciences, Monash University, Melbourne, VIC, Australia; 7 Eastern Health, Department of Respiratory and Sleep Medicine, Melbourne, VIC, Australia; 8 Eastern Health Clinical School, Monash University, Melbourne, VIC, Australia; 9 School of Agriculture and Food, Melbourne University, Melbourne, VIC, Australia; 10 School of Primary and Allied Health Care, Monash University, Melbourne, VIC, Australia

**Keywords:** Fitbit, obstructive sleep apnea (OSA), sleep duration, sleep efficiency, sleep regularity, weight

## Abstract

**Objectives:**

Weight loss is often recommended for individuals with obesity and OSA, however, weight loss is particularly hard for this population. This study investigates how weight and weight change are associated with sleep health in people with OSA.

**Methods:**

Sleep data were analyzed from participants (*n* = 28) newly diagnosed with OSA over 12-months during which time they underwent a 6-month weight-loss intervention in a step-wedge design. Sleep duration, sleep regularity, and sleep efficiency were calculated for each participant based on Fitbit data. Linear mixed-effects models were used to examine the relationships between each sleep variable and weight and weight change over the intervention period.

**Results:**

Participants were middle-aged (51 ± 10 years), living with obesity (32.6 ± 4.4 kg/m^2^), and severe OSA (AHI 30.5[26.6, 49.8] events/h). There was no association between changes in weight and sleep duration or sleep efficiency. The higher an individual's weight (kg), the more likely they were to have irregular sleep (estimate = 0.81, 95% CI [0.29, 1.32] min, and *p* = 0.002). Conversely, weight changes (kg) were not associated with changes in sleep regularity (estimate = −0.10, 95% CI [−1.51, 1.31] min, and *p* = 0.884).

**Conclusions:**

Higher weight was associated with worse sleep regularity. However, weight change was not associated with improvements in sleep regularity. Our findings demonstrate an association between weight and poor sleep in people with OSA, but the relationship is likely complex.

## Introduction

Obesity is on the rise worldwide and is associated with many adverse health conditions, including the highly prevalent sleep disorder, obstructive sleep apnea (OSA). Alarmingly, it is estimated that nearly 3/4 of adults living with obesity also have OSA (Esmaeili et al., 2025). As such, weight loss is often recommended in combination with continuous positive airway pressure (CPAP) for the treatment of comorbid obesity and OSA. Indeed, weight loss has been shown to modestly improve OSA severity (Edwards et al., 2019) and may improve some of the health effects associated with obesity. However, weight loss through lifestyle interventions such as diet and exercise can be challenging, especially in the long term.

Previous studies have reported that individuals with OSA have trouble losing weight compared with weight-matched controls without OSA (Borel et al., 2012; Janney et al., 2016). The difficulties in losing weight in individuals with OSA may be due to the bidirectional relationship between obesity and OSA (Ong et al., 2013). Obesity promotes OSA by increasing abdominal and upper airway loading, which biases the upper airway to collapse during sleep. Conversely, OSA is thought to promote obesity via the associated sleep disturbances, contributing to overeating as well as lack of energy to exercise due to fatigue, both leading to weight gain. Indeed, in the year preceding diagnosis, individuals with OSA gained, on average, 7.4 kg compared with weight-matched controls without OSA (Phillips et al., 1999). However, despite the bidirectional relationship between OSA and obesity, treating OSA with CPAP leads to a small degree of further weight gain (Drager et al., 2015), suggesting the relationship is complex.

There is a broad recognition of the relationship between disturbed sleep and weight gain in a non-OSA population (Dashti et al., 2015, 2016; Fenton et al., 2021). Specifically, several studies have shown an association between short sleep duration and obesity (Guimarães et al., 2022; Kohanmoo et al., 2024; Wu et al., 2014). More recently, the importance of sleep regularity for body weight regulation has emerged (Sletten et al., 2023). Sleep regularity refers to the intraindividual variability in sleep timing. Poor sleep regularity is associated with higher weight, weight gain, adiposity, and a blunted response to weight loss (Kobayashi et al., 2013; Ogilvie et al., 2016; Papandreou et al., 2020; Patel et al., 2014; Wong et al., 2022). In addition, several other measures of sleep health, including poor sleep efficiency, have also been associated with obesity (Ogilvie et al., 2016; Wirth et al., 2015). More directly, disturbed sleep has been shown to hinder weight-loss attempts (Kline et al., 2021; Papandreou et al., 2020). However, none of the aforementioned studies specifically evaluated these relationships in individuals with OSA.

CPAP does not address the underlying obesity that commonly co-occurs with OSA, and weight loss is particularly challenging for this group. To break the cycle of weight gain and worsening OSA, it is necessary to understand the bidirectional associations between obesity and sleep disturbances in OSA. To our knowledge no previous studies have evaluated the relationship between sleep and weight specifically in an OSA population longitudinally and understanding this relationship may allow more targeted interventions. Therefore, this study aimed to investigate how weight and weight change are associated with poor sleep health (duration, regularity, and efficiency) in people with OSA participating in a 12-month study inclusive of a 6-month weight-loss intervention following CPAP initiation. We hypothesized that higher weight would be associated with worse sleep metrics and that weight loss would be associated with improvements in those metrics.

## Materials and methods

This study involved exploratory analyses of data collected as part of the Sleeping Well Trial (Truby et al., 2019, 2022). Ethics approval for the Sleeping Well Trial was obtained by Monash Health, Eastern Health, and Monash University Human Research Ethics Committee (CF1) HREC/15/MonH/93 Ref 15357A, and the trial was prospectively registered with the Australia and New Zealand Clinical Trial Registry (ACTRN12616000203459). Participants provided written informed consent to participate in the study. Participants were included in the study if they were aged between 19 and 68 years, were initiating CPAP, had an apnea-hypopnea index (AHI) ≥ 20 events/h, and self-reported as sedentary (physical activity < 2 days per week of less than 45 min per session). Participants were excluded if they had previous treatment for OSA, bariatric surgery, inability to exercise, pregnancy, angina pectoris, atrial fibrillation, obesity hypoventilation syndrome, or an urgent need for CPAP. Participants were also excluded if their body mass index (BMI) was less than the recommended cut-off based on ethnicity-specific guidelines (24.9 kg/m^2^ for Caucasian participants, less than 23 kg/m^2^ for Indian and Chinese participants), or greater than 43 kg/m^2^. Those who were already dieting or undertaking an exercise program were excluded from participation.

### Original study design

A detailed description of the Sleeping Well Trial protocol has been previously reported (Truby et al., 2019). Briefly, the Sleeping Well trial was designed to determine whether the timing of introducing a 6-month weight loss lifestyle intervention was important for newly diagnosed patients with OSA who were initiating CPAP. The study lasted 12-months in total, and participants were randomly assigned to begin the 6-month weight-loss intervention between 1 and 6-months after enrolment (step-wedge design). Due to the step-wedged nature of the study, participants had varying pre-intervention and post-intervention times depending on when they were randomly assigned to begin the weight-loss intervention (between 1 and 6-months after the study began). The weight-loss intervention included 6-months of dietary and physical activity recommendations, along with monthly dietitian sessions. For the 6-month duration of the intervention, participants were instructed to adhere to an intermittent energy-restricted dietary protocol that comprised 5 days of energy intake of 6,300–7,500 kilojoules (kJ) per day, followed by 2 days of very low energy intake of 2,200–2,760 kJ per day. Participants were also instructed to do 3 × 30 min/week of moderate physical exercise. The physical activity instructions were a general recommendation and not a formal exercise program. As such participants self-assessed their physical activity levels using a Fitbit Flex 2 device (Fitbit, San Francisco, California). Participants had their weight measured monthly using a Seca digital weighing scale (SECA Hamburg, Germany). Weight was measured without shoes to the nearest 0.1 kg. An overnight polysomnogram (PSG) was performed on each participant either as an inpatient or at home before and after the study as previously described (Truby et al., 2019).

### Data processing

Daily sleep data was exported from each participant's web-based FitBit account for the 12-months they were enrolled in the study. Data on the length of daylight for each study day were downloaded from the Australian Government geodetic calculator (https://geodesyapps.ga.gov.au/sunrise). The Fitbit Flex 2 uses proprietary internal algorithms to determine sleep onset and sleep offset times. While the Fitbit Flex 2 is a consumer sleep tracking device it has shown to have reasonable estimates of sleep/ wake states (Cook et al., 2017; de Zambotti et al., 2018). The Fitbit derived sleep onset and sleep offset times were used to calculate sleep duration, sleep regularity and sleep efficiency. Sleep duration (total sleep time) was calculated as the time in bed minus the wake after sleep onset time, with higher values indicating longer sleep. Sleep regularity was calculated as the standard deviation of sleep duration, with higher values indicating less regular sleep (Fischer et al., 2021). Sleep efficiency was calculated as the total sleep time divided by the time in bed, expressed as a percentage, with higher values indicating more efficient sleep. All data from weekdays and weekends were combined. Only the main sleep periods were analyzed in this study due to limitations in wearables, such as the Fitbit, in detecting naps (De Zambotti et al., 2019). Any sleep onset before 20:00 or after 10:00 was considered a nap and removed before analysis. Daily sleep data were aggregated to calculate monthly means for each participant. Months with fewer than 7 days of data were removed from the analysis (Fischer et al., 2021). Participants were also removed from the analysis if they had less than 2-months of sleep data.

### Data analysis

All statistical analysis was performed using RStudio [R version 4.4.3 (2025-02-28), Inc., Boston, MA, USA]. Baseline characteristics were tested for normality using the Shapiro-Wilk test and were reported as *n* (%), mean ± standard deviation, or median (interquartile range) as appropriate. Separate linear mixed models were fitted, with sleep health measures (duration, regularity, and efficiency) as the dependent variables of interest. Participant ID was modeled as a random effect (random intercept). Weight was person-mean-centered to separate within- and between-person effects and was included in models as separate fixed effects. Specifically, the between-person weight was calculated for each participant as the mean weight averaged across all time points (i.e., “mean weight”). The within-person weight was calculated by subtracting each participant's monthly weight from their average weight (i.e., “weight change”). The within-person weight reflects the monthly variation in weight relative to the individual mean weight rather than a conventional cumulative weight loss over the study period. The within-person weight or “weight change” was chosen instead of a conventional pre-post intervention weight loss because some individuals will initially lose weight and then regain it before the end of the intervention. Between-person and within-person weight was parsed out to answer separate questions about the relationship between sleep and weight. The calculated mean weight fixed effect indicates whether someone who weighs more (or less) has poorer sleep. The weight change fixed effect indicates whether weight changes are associated with changes in sleep. Covariate-adjusted models were then fitted to assess the relationships between sleep outcomes and mean weight and weight change, while controlling for age, sex, day length, and baseline AHI. Histograms of residuals for all models were visually inspected for normality, and scatterplots of the residuals were visually inspected for heteroscedasticity. The main analysis was repeated for the sleep and weight data collected exclusively during the weight-loss intervention phase of the trial (rather than over the entire study period; see Supplementary material). Effect sizes were calculated for each model using Cohen's *f*^2^.

## Results

Sleep data from 43 participants were recorded in Fitbit. Of the 43, data from 28 participants were included in the current analysis. Of the 15 participants excluded, 3 were excluded for having less than 2-months of data, and the remaining 10 were excluded as none of their recorded months met the criterion of at least 7 days of data. In total, there were 5,339 nights of data across the 28 participants, with a mean of 191.3 days (SD 121.9) and a median of 166 days (IQR 76.5–302.3) per participant. Participants at baseline were generally middle-aged (51 ± 10 years), males (73%) living with obesity (32.6 ± 4.4 kg/m^2^) and severe OSA (AHI 30.5 [26.6, 49.8] events/h; see Table 1). Overall, three participants (10.7% of the sample) achieved 10% or more weight loss from baseline during the intervention, and 10 participants (35.7%) achieved 5% or more weight loss.

**Table 1:** Baseline participant characteristics.

Characteristic	*N* = 28
Age^*^ (years)	51 ± 10
Sex^*^ (male)	19 (73%)
Weight (kg)	97.3 ± 21.2
BMI (kg/m^2^)	32.6 ± 4.4
AHI^±^ (events/h)	30.5 [26.6, 49.8]

^*^N = 26, ^±^N = 25, BMI, body mass index; AHI, Apnea Hypopnea Index.

Data are presented as Mean ± SD, n (%), or Median [Q1, Q3] as appropriate.

### Sleep duration

**Figure 1:**
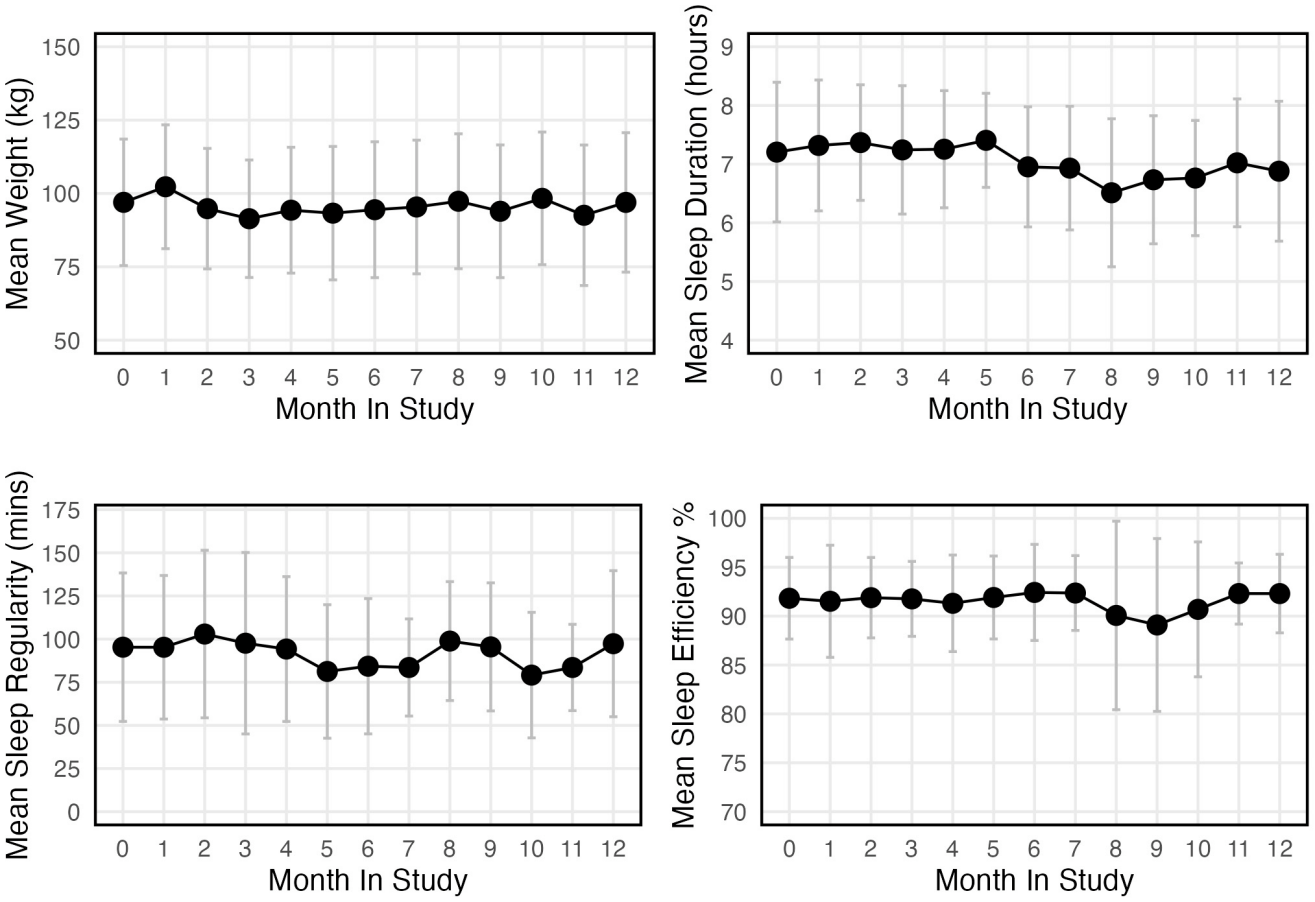
Mean weight, sleep duration, sleep regularity, and sleep efficiency for each month of the 12-month study.

The overall mean sleep duration across all participants and time points was 423 ± 59 min, equivalent to 7.05 h. The mean sleep duration for all participants for each month of the study is shown in Figure 1. In the linear mixed model, neither mean weight (estimate = −0.76, 95% CI [−1.85, 0.33] min, and *p* = 0.169) nor weight change (estimate = 0.69, 95% CI [−0.72, 2.11] min, and *p* = 0.335) was statistically associated with sleep duration (see Table 2). In the covariate-adjusted model, mean weight, weight change, age, sex, and baseline AHI were not significantly associated with sleep duration (*p* > 0.05); however, day length was negatively associated with sleep duration (estimate = −0.04, 95% CI [−0.09, −0.0003] min, and *p* = 0.048; Table 2). In other words, for every 1 min increase in day length, there was a 0.04 min decrease in sleep duration.

**Table 2:** Association between weight and sleep duration, sleep regularity, and sleep efficiency.

	Basic model											
	formula: sleep metric ~ mean weight + weight change + (1|ID)											
	Sleep duration (min)				Sleep regularity (min)				Sleep efficiency (%)			
Predictors	*B*	95% CI	β	*p*-value	*B*	95% CI	β	*p*-value	*B*	95% CI	β	*p*-value
Mean weight	−0.76	−1.85, 0.33	−0.25	0.169	0.81	0.29, 1.32	0.43	0.002	0.03	−0.05, 0.11	0.13	0.422
Weight change	0.69	−0.72, 2.11	0.03	0.335	−0.10	−1.51, 1.31	– 0.01	0.884	0.01	−0.15, 0.16	0.00	0.936
Marginal *R*^2^/conditional *R*^2^	0.059/0.785				0.157/0.489				0.017/0.596			
Overall *f*^2^	0.063				0.189				0.017			
	Covariate adjusted model											
	formula: sleep metric ~ mean weight + weight change + age + sex + day length + baseline AHI + (1|ID)											
	Sleep duration (min)				Sleep regularity (min)				Sleep efficiency (%)			
Predictors	B	95% CI	β	*p*-value	*B*	95% CI	β	*p*-value	*B*	95% CI	β	*p*-value
Mean weight	−0.32	−1.52, 0.87	−0.12	0.595	0.77	0.15, 1.38	0.42	0.015	0.03	−0.07, 0.13	0.11	0.556
Weight change	0.52	−1.09, 2.13	0.02	0.526	−0.64	−2.23, 0.95	−0.05	0.430	0.08	−0.10, 0.26	0.04	0.377
Age	0.05	−2.19, 2.28	0.00	0.967	0.14	−1.01, 1.30	0.04	0.807	−0.03	−0.21, 0.15	−0.06	0.734
Sex, male	−32.29	−86.67, 22.09	−0.53	0.243	−12.54	−40.74, 15.66	−0.31	0.382	1.46	−2.99, 5.91	0.26	0.518
Day length	−0.04	−0.09, −0.0003	−0.08	0.048	−0.02	−0.06, 0.02	−0.05	0.364	0.00	−0.00, 0.01	0.09	0.063
Baseline AHI	−0.36	−1.66, 0.94	−0.11	0.589	0.32	−0.34, 0.98	0.15	0.345	−0.03	−0.14, 0.08	−0.10	0.584
Marginal *R*^2^/conditional *R*^2^	0.101/0.789				0.161/0.524				0.035/0.639			
Overall *f*^2^	0.112				0.191				0.036			

B, unstandardized beta; CI, 95% confidence interval; β, standardized beta; AHI, apnea-hypopnea index.

### Sleep regularity

The overall mean sleep regularity across all participants and time points was 99 ± 34 min and is shown in Figure 1 for each month in the study. In the linear mixed model, there was a positive statistically significant effect of mean weight on sleep regularity (estimate = 0.81, 95% CI [0.29, 1.32] min, and *p* = 0.002; Table 2). That is, for every 10 kg higher body weight, sleep regularity increased (worsened) by 8.1 min. This significant positive association between mean weight and sleep regularity remained relatively unchanged after adjustment for covariates (estimate = 0.77, 95% CI [0.15, 1.38] min, and *p* = 0.015). Conversely, there was no statistically significant association between changes in weight and sleep regularity (estimate = −0.10, 95% CI [−1.51, 1.31] min, and *p* = 0.884; Table 2). Similarly, no significant association was observed in the adjusted model (estimate = −0.64, 95% CI [−2.23, 0.95] min, and *p* = 0.430; Table 2). Notably, none of the covariates (age, sex, day length, and baseline AHI) were significantly associated with sleep regularity (*p* > 0.05, see Table 2).

### Sleep efficiency

The overall mean sleep efficiency across all participants and time points was 91 ± 4%. The mean sleep efficiency for all participants each month in the study is shown in Figure 1. We observed no significant association between sleep efficiency and either mean weight (estimate = 0.03, 95% CI [−0.05, 0.11] min, and *p* = 0.422) or weight change (estimate = 0.01, 95% CI [−0.15, 0.16] min, and *p* = 0.936; Table 2). This observation was not altered after adjustment for (age, sex, day length, and baseline AHI; Table 2).

## Discussion

This study aimed to examine the associations between body weight and weight change and several markers of sleep health (i.e., sleep duration, sleep regularity, and sleep efficiency) in people with OSA undergoing a weight loss intervention who were initiating CPAP treatment. Our findings indicate that those with higher body weight at baseline had poorer sleep regularity (greater night-to-night variability in sleep duration), even after controlling for age, sex, day length, and baseline AHI. However, changes in weight over the duration of the intervention were not associated with statistically meaningful changes in sleep regularity. Furthermore, there was no association between weight or weight change and sleep duration or sleep efficiency. Collectively, our results suggest that heavier individuals with OSA have more irregular sleep even when treated with CPAP, however, weight change of the magnitude seen in this trial may be inadequate to improve sleep regularity.

Our finding that increased weight was associated with poorer sleep regularity in an OSA population, while not directly proving a bidirectional relationship between weight and OSA, supports the plausibility of this model. Irregular sleep can disrupt the circadian system, leading to circadian misalignment, which can alter satiety hormones, decrease energy expenditure, and promote unhealthy eating, all of which may contribute to weight gain (Chaput et al., 2023). Weight gain then places excess loading on the abdomen and upper airway, which biases the upper airway to collapse during sleep, worsening OSA. These findings may help explain why people with OSA have more difficulty losing weight than those without OSA (Borel et al., 2012; Janney et al., 2016).

Contrary to our hypothesis, we found that weight change during the intervention was not associated with changes in sleep regularity. The lack of association could be because the small changes in weight in this study were not large enough to result in significant changes in sleep regularity. The intervention led to only a minority (three participants) to achieve the 10% weight loss typically considered a critical threshold for improving metabolic outcomes (Ryan and Yockey, 2017). It could also reflect constraints imposed by lifestyle factors, such as social jetlag and family and work obligations. Future research should determine whether weight-loss interventions that typically yield larger reductions in weight (e.g., bariatric surgery or GLP-1 agonist therapy) are associated with improvements in sleep regularity in people with OSA. Furthermore, the 12-month study duration may not have been long enough to detect changes in sleep regularity.

To our knowledge, the current study is the first to explore the link between weight and sleep regularity among participants with OSA. However, a previous study examined sleep regularity and its association with hypertension in individuals with/without OSA (Sansom et al., 2024). Those with mildly irregular or severely irregular sleep were more likely to have OSA than regular sleepers. Furthermore, those with OSA who were severely irregular sleepers were at a higher risk for hypertension, but those with OSA who were regular sleepers or mildly irregular sleepers were not. The authors of this paper suggest that sleep regularity could be a modifiable target to reduce the risk of hypertension in people with OSA.

The current study found no association between weight and sleep duration or sleep efficiency. The lack of association may be because, despite having OSA and obesity, participants in this study generally had a relatively good sleep duration and sleep efficiency to begin with. The mean monthly sleep duration in this study was approximately 7 h. In the MESA study, higher BMI, waist circumference, and body fat percentage were found among individuals who slept less than 6 h per night compared with those who slept 7–8 h per night (Ogilvie et al., 2016). Similarly, mean monthly sleep efficiency in this current study was generally greater than 90% which is considered sufficient. Conversely, while there is no official cut-off of good vs. poor sleep regularity, the mean monthly sleep regularity was well-above 1 h. (He et al. 2015) found that a 1 h increase in sleep regularity was associated with a 170 kcal increase in daily energy intake, suggesting that poor sleep regularity may contribute to weight gain by increasing calorie consumption. Overall, the findings suggest that our study population had relatively healthy sleep duration and sleep efficiency, which may have limited the ability to detect associations with weight and weight change, but sleep regularity was poor.

A strength of our analysis was the longitudinal design, which has been a limitation of previous studies. However, this study has several limitations. Firstly, the lack of weight loss in this study may have attenuated the findings. Given that weight-loss via lifestyle interventions is often modest, future studies should investigate the longitudinal associations between sleep and weight in larger OSA populations undergoing treatment with tirzepatide, which has been shown to result in substantial weight loss (Malhotra et al., 2024). In addition, the sample size was small due to non-adherence with regular Fitbit use, the small sample size limits the statistical power and generalizability of the findings in this study. While most participants wore the Fitbit for some portion of the study, it was often not enough (at least 7 days in a month) to warrant inclusion in the current analysis. The reason for non-adherence is unknown. However, it may have been due to the overwhelming number of new things participants were asked to do as newly diagnosed patients with OSA for this study (CPAP, diet, and exercise). Due to the low sample size, we were likely underpowered to detect true clinical differences. Indeed, based on our observed effect sizes, *post-hoc* power calculations indicate that our retrospective analysis was underpowered to detect small to moderate effects for sleep duration (1–β = 0.063) and sleep efficiency (1–β = 0.017) and only slightly underpowered for sleep regularity (1–β = 0.189). The study was also limited by the use of the Fitbit Flex 2, a consumer sleep tracking device which is limited in detecting sleep/wake states in comparison to polysomnography (Lee et al., 2025). Another limitation of this study was the use of only one type of weight metric (weight). While body fat and waist circumference were measured at some time points in this study, the measurements were not regular enough to be included in the analysis.

In conclusion, the current study found that higher weight was associated with poorer sleep regularity, as estimated by Fitbit, in people with OSA during a 12-month CPAP and weight-loss intervention. However, weight loss was not associated with significant changes in sleep regularity, which may be a limitation given the modest weight loss and the relatively good sleep quality observed in this cohort. Our findings support a complex bidirectional relationship between weight and OSA. Future studies should investigate whether improving sleep beyond CPAP alone can help individuals with OSA achieve greater weight loss.

## Data Availability

The raw data supporting the conclusions of this article will be made available by the authors upon reasonable request.
