# Supplementary material for: Weight and sleep health in OSA: exploring their link
Source: Front Sleep. 2026 May 18;5:1828583. doi: 10.3389/frsle.2026.1828583 (PMC13222836; doi:10.3389/frsle.2026.1828583)
Supplement: Supplementary file 1 [file Supplementary_file_1.docx]

# **Weight and Sleep Health In OSA: Exploring Their Link**

Caroline J. Beatty, Shane A. Landry, Dwayne L. Mann, Simon A. Joosten, Kaitlin Day, Maxine P. Bonham, Denise M. O’Driscoll, Alan Young, Ladan Ghazi, Chiara Murgia, Terry P. Haines, Garun S. Hamilton^#^, Bradley A. Edwards^#^*

^#^ Equal Contributions

*** Corresponding author: bradley.edwards@monash.edu

**Supplementary Results**

**Repeat of Main Analysis Using Data Exclusively from the Weight Loss Intervention Phase of the Study**

The primary analysis has been repeated using data exclusively from the participants' time in the weight-loss intervention phase of the study, which lasted 6 months of the 12-month study. Due to the step wedge design of the study, participants began the intervention at varying times from months 1-6 (i.e., some started in month 2 of 12 of the study, and others began in month 6 of 12 in the study). The weight-loss intervention included monthly sessions with a dietitian and recommendations for physical activity. Sleep data from 24 of 28 participants were included in this sub-analysis. Exclusion of 4 participants was due to either an insufficient or complete lack of Fitbit data recorded during the weight-loss intervention phase of the study.

1. ***Sleep Duration***

The mean sleep duration for all participants in each month of the intervention is shown in Figure S1. In the linear mixed model, neither mean weight (estimate = -0.49, 95% CI [1.77 – 0.79] mins, p = 0.448) nor weight change (estimate = 0.80, 95% CI [-1.22 – 2.81] mins, p = 0.434) was statistically associated with sleep duration (see Table S1). In the covariate-adjusted model, mean weight, weight change, age, sex and baseline AHI were not significantly associated with sleep duration (p > 0.05).

1. ***Sleep Regularity***

The mean sleep regularity for all participants in each month of the intervention is shown in Figure S1. In the linear mixed model, neither mean weight (estimate = 0.58, 95% CI [-0.02 – 1.19] mins, p = 0.059) nor weight change (estimate = 0.47, 95% CI [-2.33 – 3.27] mins, p = 0.739) was statistically associated with sleep regularity (see Table S1). In the covariate-adjusted model, mean weight, weight change, age, sex and baseline AHI were not significantly associated with sleep regularity (p > 0.05).

1. ***Sleep Efficiency***

The mean sleep efficiency for all participants in each month of the intervention is shown in Figure S1. In the linear mixed model, neither mean weight (estimate = 0.07, 95% CI [-0.05 – 0.19] mins, p = 0.247) nor weight change (estimate = -0.03, 95% CI [-0.31 – 0.25] mins, p = 0.852) was statistically associated with sleep efficiency (see Table S1). In the covariate-adjusted model, mean weight, weight change, age, sex, and baseline AHI were not significantly associated with sleep efficiency (p > 0.05); however, day length was significantly associated with sleep efficiency (estimate = 0.01, 95% CI [0.00 – 0.02] mins, p = 0.009).


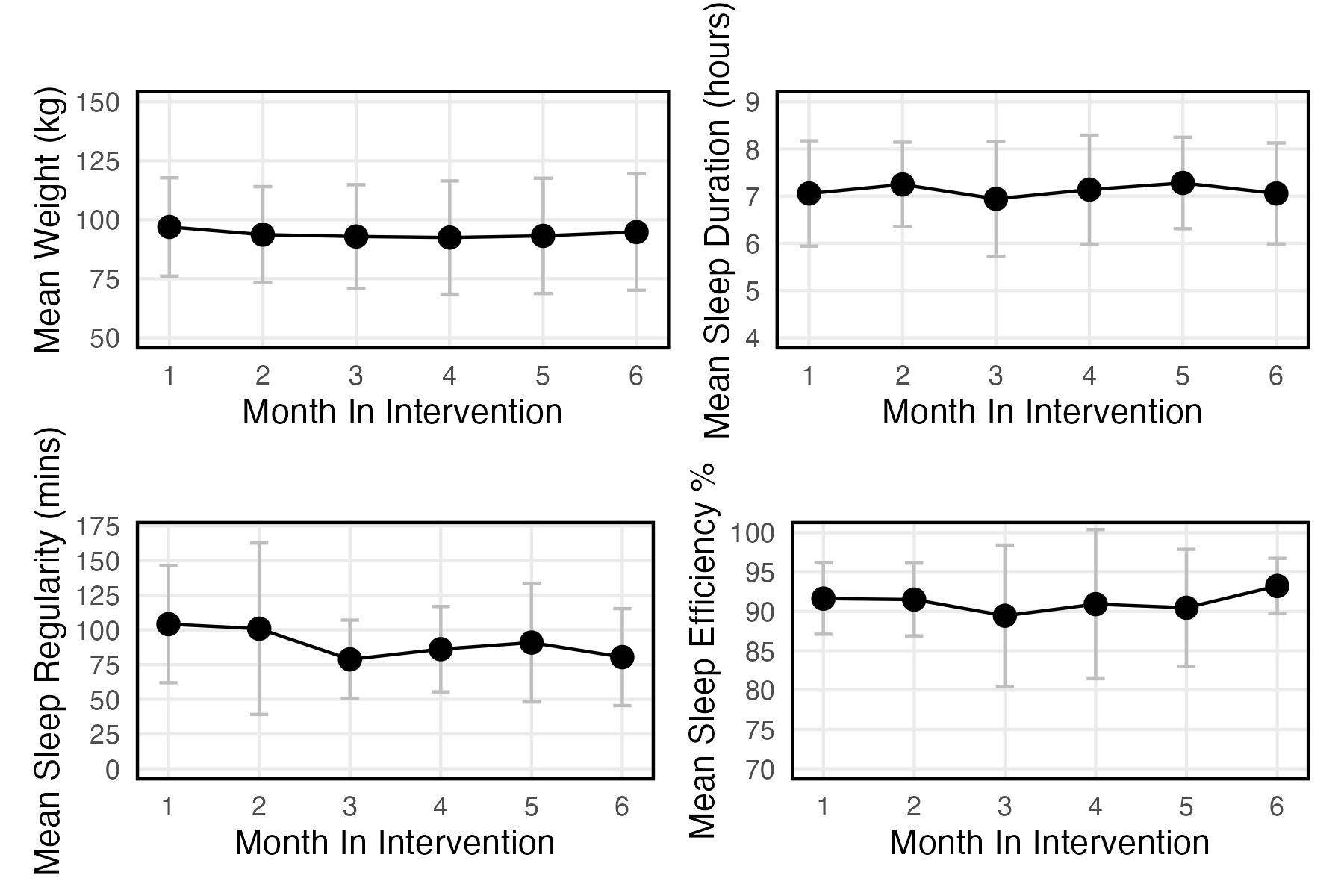


Figure S1: Mean weight, sleep duration, sleep regularity and sleep efficiency for each month of the 6-month study.

Table S1: Association Between Weight and Sleep Duration, Sleep Regularity and Sleep Efficiency During the Weight Loss Intervention Phase of the Study

|  | **Basic Model**  formula: sleep metric ~ mean weight + weight change + (1\|ID) | | | | | | | | | | | |
| --- | --- | --- | --- | --- | --- | --- | --- | --- | --- | --- | --- | --- |
|  | *Sleep Duration (min)* | | | | *Sleep Regularity (min)* | | | | *Sleep Efficiency (%)* | | | |
| **Predictors** | **B** | **95% CI** | **β** | **p-value** | **B** | **95% CI** | **β** | **p-value** | **B** | **95% CI** | **β** | **p-value** |
| Mean Weight | -0.49 | -1.77, 0.79 | -0.17 | 0.448 | 0.58 | -0.02, 1.19 | 0.29 | 0.059 | 0.07 | -0.05, 0.19 | 0.23 | 0.247 |
| Weight Change | 0.80 | -1.22, 2.81 | 0.03 | 0.434 | 0.47 | -2.33, 3.27 | 0.03 | 0.739 | -0.03 | -0.31, 0.25 | -0.01 | 0.852 |
| Marginal R^2^/ Conditional R^2^ | 0.027 / 0.851 | | | | 0.080 / 0.373 | | | | 0.051 / 0.714 | | | |
| Overall f^2^ | 0.027 | | | | 0.087 |  |  |  | 0.054 | | | |
|  | **Covariate Adjusted Model**  formula: sleep metric ~ mean weight + weight change + age + sex + day length + baseline AHI + (1\|ID) | | | | | | | | | | | |
|  | *Sleep Duration (min)* | | | | *Sleep Regularity (min)* | | | | *Sleep Efficiency (%)* | | | |
| **Predictors** | **B** | **95% CI** | **β** | **p-value** | **B** | **95% CI** | **β** | **p-value** | **B** | **95% CI** | **β** | **p-value** |
| Mean Weight | -0.11 | -1.54, 1.31 | -0.04 | 0.874 | 0.52 | -0.19, 1.22 | 0.26 | 0.148 | 0.06 | -0.08, 0.19 | 0.18 | 0.414 |
| Weight Change | 0.49 | -1.79, 2.77 | 0.02 | 0.668 | -0.18 | -3.26, 2.90 | -0.01 | 0.910 | 0.16 | -0.14, 0.47 | 0.06 | 0.291 |
| Age | -0.02 | -2.48, 2.45 | -0.002 | 0.990 | -0.21 | -1.45 , 1.02 | -0.05 | 0.734 | -0.02 | -0.25, 0.22 | -0.03 | 0.873 |
| Sex, male | -17.57 | -79.94, 44.80 | -0.28 | 0.577 | -1.95 | -33.98, 30.08 | -0.05 | 0.904 | 3.26 | -2.70, 9.23 | 0.49 | 0.280 |
| Day Length | -0.02 | -0.09, 0.05 | -0.03 | 0.573 | -0.04 | -0.13, 0.04 | -0.11 | 0.333 | 0.01 | 0.00 ,0.02 | 0.20 | **0.009** |
| Baseline AHI | -0.59 | -2.05, 0.86 | -0.19 | 0.421 | 0.30 | -0.42, 1.02 | 0.14 | 0.405 | -0.05 | -0.19, 0.09 | -0.15 | 0.474 |
| Marginal R^2^/ Conditional R^2^ | 0.066 / 0.861 | | | | 0.090 / 0.406 | | | | 0.100 / 0.756 | | | |
| Overall f^2^ | 0.071 |  |  |  | 0.099 |  |  |  | 0.111 |  |  |  |
| B = unstandardized beta, CI = 95% confidence interval, β = standardized beta, AHI = apnea-hypopnea index | | | | | | | | | | | | |
